# The Biomechanical Profile of an Osseo-Integrated Rectangular Block Implant: A Pilot In Vivo Strain Analysis

**DOI:** 10.3390/bioengineering9090425

**Published:** 2022-08-30

**Authors:** Efthimios Gazelakis, Roy B. Judge, Joseph E. A. Palamara, Mohsin Nazir

**Affiliations:** Melbourne Dental School, University of Melbourne, Parkville, VIC 3010, Australia

**Keywords:** rectangular block implant, osseo-integration, masticatory load, stress concentration, strain generation

## Abstract

*Aim:* To load-test the osseo-integrated rectangular block implant (RBI), measure the generated cortical peri-implant strains, and relate these findings to known human physiological parameters. *Materials and methods*: Two RBIs were placed into the posterior mandibular saddle in a mature greyhound dog and allowed to osseo-integrate. The half mandible (implants in situ) was mounted in a servohydraulic system. Four triple-stacked rosette gauges were placed cortically (mesial, distal, buccal, and lingual). A modified ISO-14801 protocol was used (1000 N, 30^0^, 2 Hz, 1 h) and the generated principal strains (ep, eq) and their angular orientations (F), were calculated. *Results:* (1) Bucco-lingual “horizontal” dimension: dominant “horizontal” compressive stresses were on the lingual aspect and “horizontal” tensile stresses on the buccal aspect. The buccal cortex was elastically tensile-stretched, while the lingual cortex was elastically compressed. (2) Bucco-lingual “vertical” dimension: dominant vertical torsional stresses were oriented buccally and apically, with an overall buccally inclined torsional effect. This was also evidenced on the lingual aspect, where there remained high torsional rotation elements (high F and e2). (3) Mesio-distal “horizontal” dimension: dominant torsional stresses oriented as a distal-lingual “counter-clockwise” rotation. *Conclusions:* The applied off-axial loads generated a heterogeneous pattern of bucco-lingual and mesio-distal cortical strains, both vertically and horizontally. The short dimensioned osseo-integrated RBI design appeared to biomechanically withstand the applied loads and to maintain the strains generated to levels that were within physiological limits. More studies and statistical analyses are needed to confirm these findings.

## 1. Introduction

Short implant-supported prosthodontic success for the resorbed posterior saddle has been reported as high [1]. This problem is especially evident in the mandibular posterior saddle, where occlusal loads are high [2] and where, after many years, the ridge is resorbed. The importance of implant design in addressing functional demands and reducing stress concentration has been extensively discussed [3,4]. This research project poses a novel design of dental implant, the rectangular block implant (RBI). It is specifically designed to address the needs of patients with this clinical problem. It represents a new concept in implant design and force distribution [5].

Occlusal overload has been defined as a load that exceeds the load-bearing capacity of the implant and its prosthesis, resulting in the biological failure of osseo-integration and the mechanical failure of the prosthesis [6].

Despite the suggestion that occlusal overload may serve as a major contributing factor to prosthetic failure [7,8,9], various clinical and animal studies have provided conflicting evidence regarding its negative impact on implant osseo-integration [10,11,12].

Based on clinical observations, late implant failures have been related to heavy occlusal contacts and/or parafunctional habits [13,14,15,16,17], excessive cantilever lengths [14,18,19,20], the significant deviation of the prosthesis from the implant axis, and increased crown–implant ratios [21]. Conversely, other clinical studies have disagreed with the association between occlusal load and increased crestal bone loss [22,23,24,25,26,27,28].

Strain gauge analysis has been extensively applied to bone [29] and the peri-implant cortical elements of osseo-integrated fixtures in an effort to characterize the impact of the loads applied to the adjacent bone [2]. The importance of these endeavors is exemplified by work [30] which has first established that bony structure adapts through remodeling processes to maximally withstand applied loads and secondly, clarified strain levels as determinants of the capacity of bone to undergo such physiological adaptation. Frost [30,31,32] defined adaptive bone physiological limits where remodeling begins at approximately 500 με and ceases at approximately 3000 με, beyond which a pathological state exists.

This endeavor is not designed to replace cylindrical implant designs that currently exist. It is aimed at providing a specialized implant fixture for a particular clinical application, that is, the restoration of the resorbed alveolar ridge. The RBI presents a novel approach in that its longitudinal axis is along the mesial-distal length of the remaining ridge, which is the very dimension in which nature preserves the maximal remaining bone volume, while its flat surfaces provide an even force distribution and maximizes surface area.

The aim of this article is to qualitatively analyze the biomechanical characteristics of the osseo-integrated RBI by determining the peri-implant cortical strains generated through the application of high (1000 N) unfavorably angulated loads that simulate extreme masticatory force. It is a qualitative analysis, in supplementation of the post-integration mechanical testing of the osseo-integrated RBI (mechanical push and pull tests) that has already been performed [5].

## 2. Materials and Methods

### 2.1. The Rectangular Block Implant

For details of design, readers are referred to patents: Europe and the United Kingdom (EP 32911759 B1; Australia (2016257149 B2). The manufacture of the RBI has been described in an earlier publication [5].

### 2.2. Animal Surgery and Ethics

All procedures were performed under Animal Ethics approval (*see below*). The approval for this in vivo animal model and its selection was based on its ability to mimic the human resorbed alveolus after creation of edentulous posterior regions.

The surgical protocol evaluation and clinical testing were achieved through the placement of 2 RBIs into a mature male greyhound dog in a prepared (left side) posterior edentulous mandibular saddle. This animal model was chosen on the basis of its thin mandibular structure and underlying inferior alveolar nerve neurovascular bundle, closely simulating that of the human structure in the edentulous state. The details of the surgical aspect of implant placement, including anesthesia, have been described elsewhere [5].

Ethical constraints in this study mitigated against the use of greater numbers of animals and mandated the use of the minimal possible number. As a result, one animal only was available for this component of the RBI’s biomechanical analysis.

The animal experimentation protocol (including the research question, key design features, and analysis plan) was prepared prior to the commencement of this study and submitted for approval (and retained) by the Animal Ethics Committee at the University of Melbourne. Upon approval, the conduct and reporting of the study were executed in accordance with the principles of Enhancing the Quality and Transparency Of health Research (EQUATOR), which are embodied by [33] as applied to animal experimentation: Animal Research Reporting In Vivo Experiments (ARRIVE).

### 2.3. Osseo-Integration

This was assessed clinically at second-stage surgery exposure, at which time the animals were sedated, and then euthanized through administration of 20 mL of sodium pentobarbitone (325 mg/mL) into the right cephalic vein.

Resonance frequency analysis (RFA) (Osstell, W & H Pty Ltd., Sydney, Australia, Smart Peg #47) and torque tests (torque wrench: Nobel Biocare, Sydney, Australia) applied through a cylindrical healing abutment were performed. Successful clinical osseo-integration was deemed to have been achieved if the implant produced Osstell readings over 80 from all directions (mesial, distal, lingual, and buccal), as well as five torque test levels of over 35 Ncm.

### 2.4. Embedding

After second-stage clinical testing, the mandible was carefully dissected, de-fleshed, and sectioned in two through the midline to isolate the half mandible arch into two, containing the two implants.

**Figure 1:**
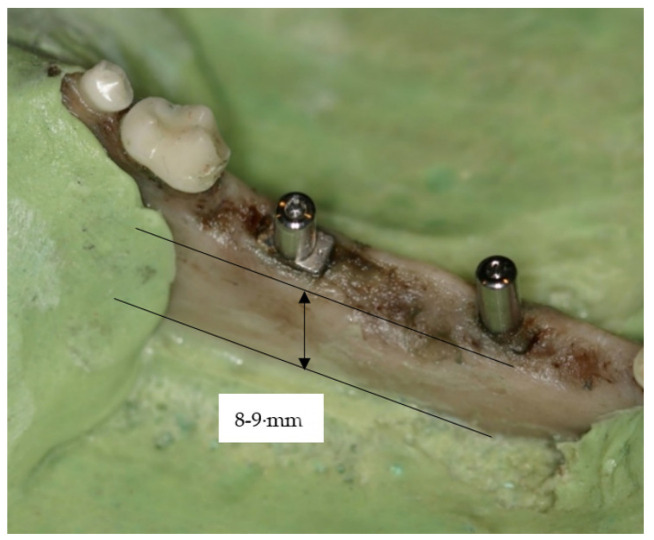
Embedded half mandible in acrylic, leaving the exposed alveolus and integrated implants.

The left half of the mandible was embedded in acrylic material (Vertex Trayplast, Vertex-Dental BV, The Netherlands), such that it was rigidly stabilized. The acrylic was ground and sanded to produce a platform with a flat base. The mandibular section was kept as one long piece as much as practicably possible (Figure 1).

The embedment of the bone was limited so as to expose the entire implanted region. Buccal and lingual bone was left exposed to a depth (8–9 mm) such that it exceeded the depth of the implants (5.5 mm).

The mesial implant had passed both tests at the second stage and, hence, was used for this component of the study. The distal implant was left undisturbed in situ throughout the remaining course of this experiment and remained as a “spare”.

### 2.5. Prosthetic Crown and the Force Application

A titanium (Grade V) prosthetic abutment designed to engage the unique RBI internal geometry was prepared and inserted into the mesial RBI at 35 Ncm.

**Figure 2:**
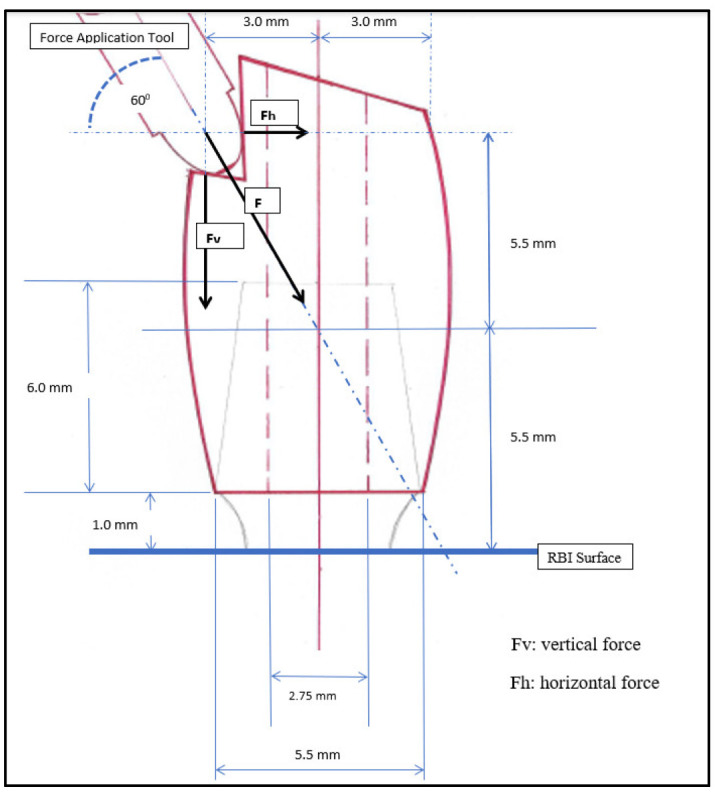
Prosthetic tooth design (bucco-lingual section): the point of force application, F, is on the lingual aspect.

A prosthetic test crown (Cobalt Chrome alloy) was designed (Figure 2) with a central hollow shaft for abutment screw access and prepared (Figure 2) with a grooved step placed on the lingual aspect for a buccally oriented off-axial load application. The crown was trial fitted to ensure complete and stable seating on the prosthetic abutment.

After confirmation of fit, Cavit (Dentsply, Australia) temporary material was placed over the opening of the screw shaft of the prosthetic abutment and the crown was cemented into position with Fuji Plus (GC Corporation, Japan) glass ionomer luting cement. Excess material was removed and a period of at least 24 h was allowed for complete setting.

**Figure 3:**
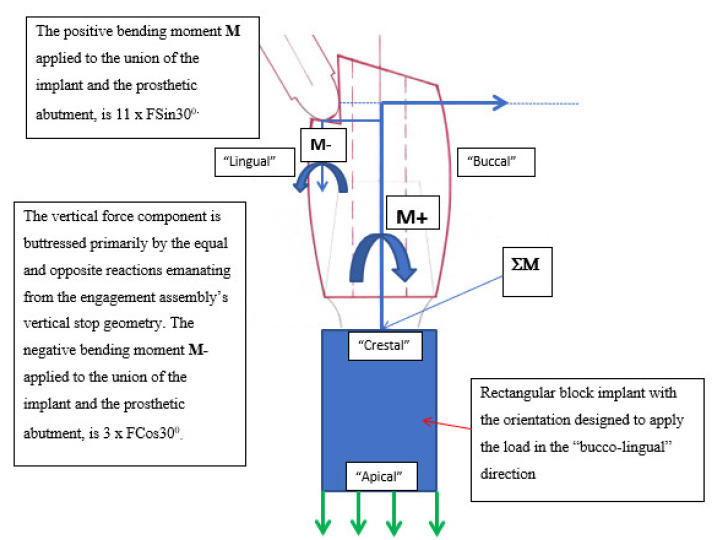
Test assembly: crestal region moments generated. The residual axial forces (green arrows) would be expressed apically.

Figure 3 depicts the resultant moments from the force application. It is noted that M+ > M−; hence, ΣM is positive (i.e., 2.9 F) but less than M+. This was compounded by the fact that the anti-rotational geometry of the assembly had a much more definitive vertical stop design than horizontal limitation. This was a natural physical consequence of the axial path of engagement between the prosthetic abutment and its underlying implant geometry.

These calculations pertained primarily to the crestal regions and have been based on the assumption of the pivotal point being at the crestal surface (i.e., at 11 mm). It should be clarified, however, that, after factoring in the effect of the axial force component, this pivotal point would be transferred deeper into the mandible (depending on the relative stiffness of the cortico-cancellous architecture). The exact position of this remained unknown.

### 2.6. Strain Gauges

The gauges used were of a stacked rosette design: miniature three-element 45-degree rosettes ((WA06030WR120) Measurements Group Inc., Raleigh, NC, USA). The configuration used in the current project was that of a quarter bridge (that is, 3 precise resistors and 1 active in each gauge).

The stacked rosette gauge ensured that all three-gauge elements were above the portion of the region being examined. Each of the grids was at 45 degrees to the other. The area of overlap defined the portion of the cortex being examined.

**Figure 4:**
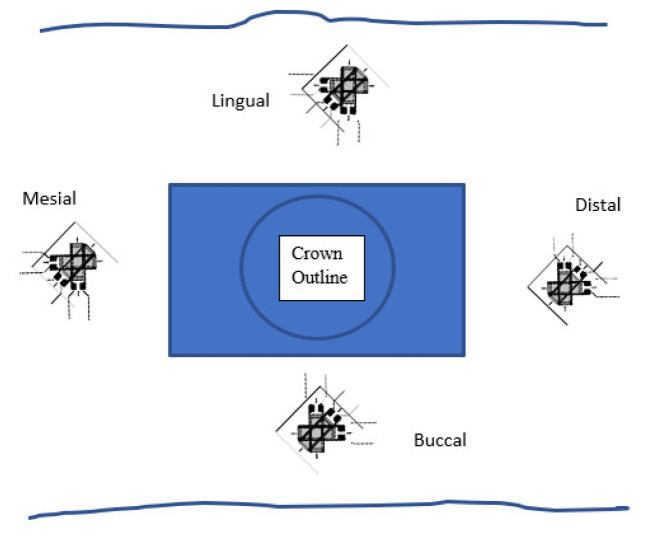
Strain gauge distribution: plan view. Note that this plan view does not show that the buccal and lingual gauges were “vertically oriented” with respect to the mesial and distal (crestal “horizontally oriented”) gauges.

Strain gauges were then adhered onto the mesial crestal, distal crestal, lingual vertical, and buccal vertical bony cortices (M Bond 200 Adhesive Kit, Measurements Group Inc., Raleigh, NC, USA) (Figure 4).

The 45^0^ triple-stacked rosettes yielded data produced by each double-wired output channel (i.e., 6 wires, 3 channels). These channels (0–5) were ascribed the strain values ε1, ε2, and ε3 in the following “flat-plan” configuration.

**Figure 5:**
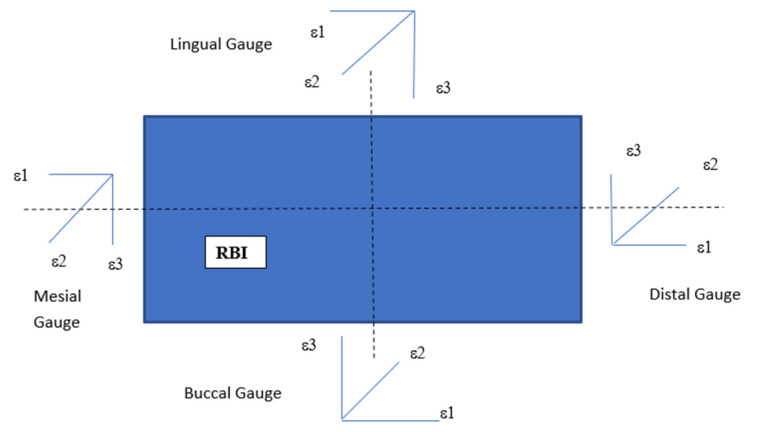
Orientation of gauge leads with respect to the implant surfaces. The orientation of the mesial and distal rosettes and their leads was such that ε1 was perpendicular to the adjacent surface of the implant, ε3 was parallel to this surface, while that of the buccal and lingual gauges was such that ε3 was perpendicular to the adjacent surface of the implant and ε1 was parallel to this surface; ε2 was at 45^0^ to each of these in each case.

The mesial and distal gauges were in the “horizontal-crestal” plane parallel to the “occlusal plane”. The buccal and lingual gauges were, in their respective “vertical” planes, perpendicular to the crestal surface. These channels were ascribed the strain values ε1, ε2, and ε3 in the following “flat-plan” configuration (Figure 5).

A series of secondary terminals were bonded adjacent to each of the gauges in an area not being investigated. The secondary terminal prevented any strain transferring to the gauge from accidental pulling of the wires. The lead wires were connected to the secondary terminals using fine color-coded Teflon-encased wires (Miniature PTFE Wire (H-UT 3607 T F) Habia Cable Asia Ltd., Swedish Branch, Soderfors, Sweden).

The wires from the secondary terminals that were a larger gauge cable were used to connect to the data acquisition system (Data Logger, CPE Systems, Victoria, Australia). The strain gauges were connected to a Wheatstone signal conditioning module (SCC-SG03 National Instruments Corp., Austin, TX, USA) housed in a configurable connector unit (SC-2345 National Instruments Corp., Austin, TX, USA).

The outputs went directly to this system. The data were then processed and collated using the lab view program supplied with the acquisition system. Computer software: (Lab View 7 Express, National Instruments Corp., Austin, TX, USA). An initial measurement of 120 Ω ensured that the gauge was functional and ready to be tested.

### 2.7. The Mounted Set-Up

**Figure 6:**
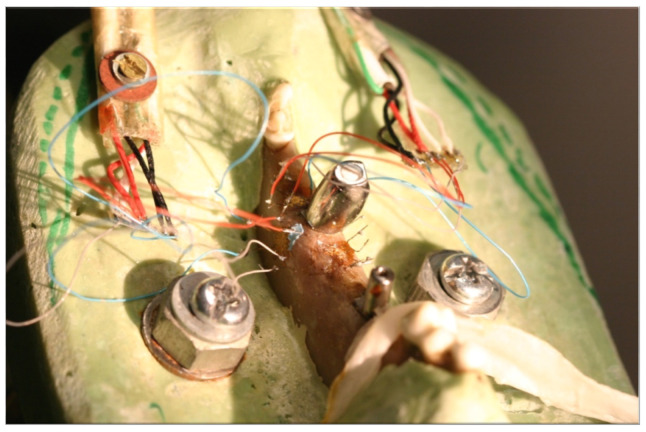
Buccal and mesial wires connected; space limitation meant that only 2 gauges at a time could be used. This image shows the mesial and buccal gauges connected to the system.

The acrylic embedding was bolted onto a brass plate attached to the base of the servo-hydraulic platform with an angulated face of 30^0^ from the vertical (Figure 6). The bone was kept moist with saline through the use of a paper towel wick to avoid desiccation.

The first test run engaged the mesial and buccal gauges, while the second engaged the lingual and distal. Uni-axial sinusoidal cyclic loading was at a possible frequency range of 3–5 Hz for a total of up to 3 × 10^4^ cycles, utilizing closed-loop servo-hydraulics (MTS 810, Materials Test Systems Corporation, Eden Praire, MN). This frequency and cycle number was chosen to expedite the test process, without deviating significantly from a low frequency–high cycle regime. The final ε value for each channel was determined through the mean difference between maximal and minimal detections of surface deflection to yield positive (tension) and negative (compression) values.

## 3. Results

### 3.1. Channel Data

**Scheme 1:**
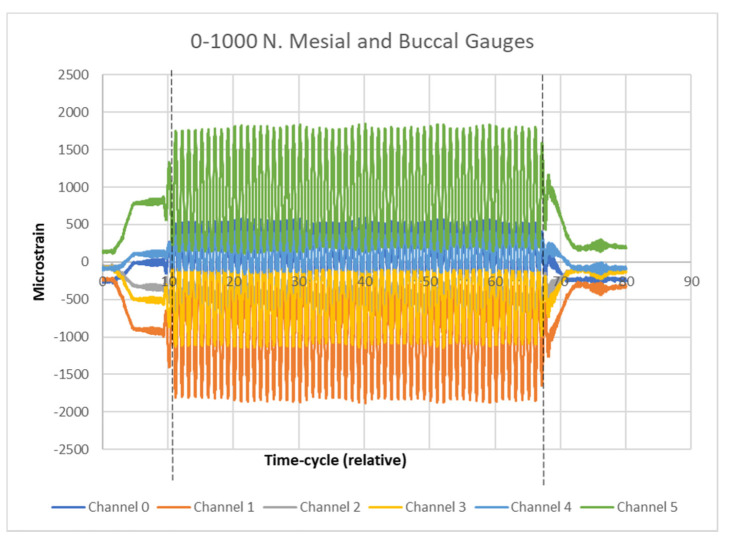
For each gauge, there was a mixture of compressive (negative) and tensile (positive) strains. Mesial gauge: Channel 0 (ε1) positive, Channel 1 (ε2) negative, Channel 2 (ε3) negative. Buccal gauge: Channel 3 (ε1) negative, Channel 4 (ε2) positive, Channel 5 (ε3) positive.

**Scheme 2:**
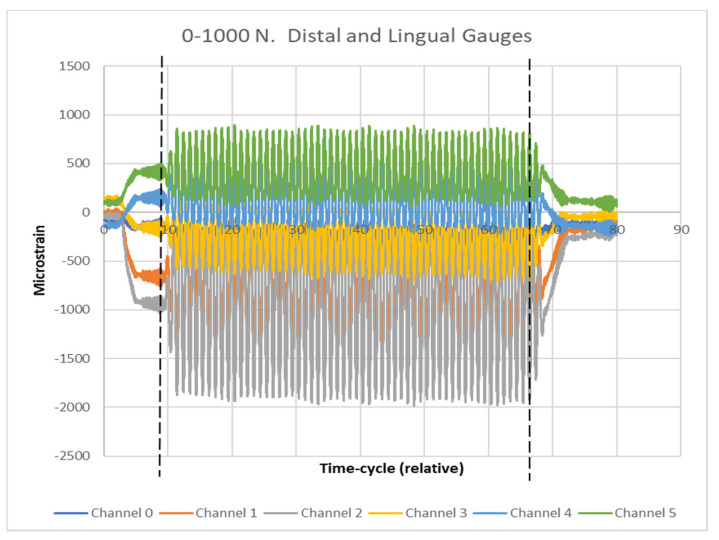
The absolute strain values (relative amplitudes for 01000 N load). The distal gauge results were all in compression (negative). Distal gauge: Channel 0 (ε1) negative, Channel 1 (ε2) negative, Channel 2 (ε3) negative. For the lingual gauge there was a mixture of compressive (negative) and tensile (positive) strains. Lingual gauge: Channel 3 (ε1) negative, Channel 4 (ε2) positive, Channel 5 (ε3) positive.

Scheme 1 and Scheme 2 depict the sinusoidal patterns of the gauge channels. The mean difference (and standard deviation) between maxima and minima for each of the channels was calculated. The results are depicted in Table 1.

### 3.2. Principal Strains

Using a “strain-transformation” relationship, the principal strains from the rosette gauge were calculated (Vishay Measurements Group, NC, USA: Technical Note-515) (Table 2). This is expressed as the two principal strains (perpendicular to each other) and their angular orientation to the reference grid. Since the direction of the principal strain is unknown, the angle (θ) is from the direction of the principal axis εp or εq (the determination of which is given below) to a specified strain gauge reference grid (either ε1 or ε3) according to:

(a) If ε1 > ε3, then ϕ*p*,*q* = ϕ*p*: the reference is from ε1 to εp.
(b) If ε1 < ε3, then ϕ*p*,*q* = ϕ*q*: the reference is from ε3 to εq.
(c) If ε1 = ε3 and ε2 < ε1, then ϕ*p*,*q* = ϕ*p* = −45°.
(d) If ε1 = ε3 and ε2 > ε1, then ϕ*p*,*q* = ϕ*p* = +45°.
(e) If ε1 = ε2 = ε3, then ϕ*p*,*q* is indeterminate (equal bi-axial strain).

Where ε2 values are ascribed as large, this was:

(i) Relative to the associated *absolute* ε1 and ε3 readings for that output;
(ii) Indicative of a high torsional element affecting that peri-implant cortex.

**Figure 7:**
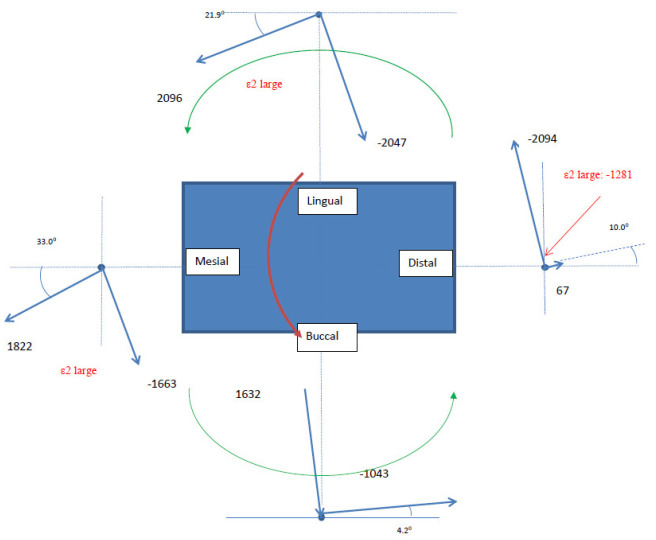
Principal strains and their orientations (not drawn to scale): where, indicated in red, the value of ε2 was of a relatively large magnitude, and this has been included to be indicative of a high torsional element. The green circular arrows represent the overall “horizontal” torsional (rotatory) effect on the RBI. Horizontally: the mesial and distal gauges now combined to show an overall counter-clockwise torsion (green arrows). Vertically: there was a strong push to the buccal: the overall trend was a buccally oriented torsion (red arrow).

The overall plan view of these principal strains with respect to their proximal implant surface is depicted in Figure 7.

### 3.3. Observational Summary

Mesio-distally: the large ε2 values represented strong torsional elements, and the distal counter-clockwise rotation was evident (as defined in the current plan view depiction of the block).

Bucco-lingually: the compressive strains on the lingual aspect were evident, as well as large tensile strains on the buccal aspect. This was especially evident on the buccal aspect, where there was a very low Φ value, yielding essentially the buccal tensional load and the rotational element.

On the lingual aspect, however, there remained high torsion rotational elements (high Φ and ε2), giving a complex picture of lingual compression, a buccally directed torsion, and a block torsion in a counter-clockwise rotation.

## 4. Discussion

### 4.1. Measured Cortical Strains

Although the data generated were collected in mesio-buccal and then disto-lingual pairs, the results of these experiments were analyzed, by comparing the principal strains and angular orientations of the gauge readings, as paired mesio-distal and bucco-lingual outputs. The combined effects of the resultant paired angulated tensile-compressive strains highlighted a complex array of results, where dominant patterns encompassed:

(a) In the buco-lingual horizontal dimension: dominant “horizontal” compressive stresses on the lingual aspect, coupled with “horizontal” tensile stresses on the buccal aspect.
(b) In the bucco-lingual “vertical” dimension: dominant vertical torsional stresses oriented buccally and apically.
(c) In the mesio-distal “horizontal” dimension: dominant torsional stresses oriented as a distal-buccal “counter-clockwise” rotation.

The bucco-lingual and the mesio-distal aspects will now be discussed in turn and then as a composite picture.

### 4.2. Tensile and Compressive Elements in the Bucco-Lingual (Horizontal) Dimension

The ramifications of this pattern were two-fold.

(i) Horizontally. In the horizontal bucco-lingual aspect, this could be seen as an elastic deformation of the body of the mandible. The deformation of the lingual cortex would have resulted in concavity, while that of the buccal cortex would have resulted in convexity. This equated to a three-point bending configuration.

**Figure 8:**
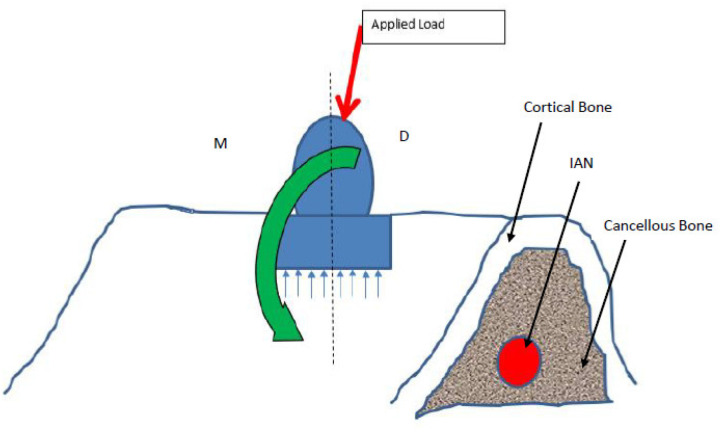
Vertical bucco-lingual torsional effect: buccal view. The red arrow depicts the applied force, while the vertical green arrow depicts the bucco-lingual “vertical” resultant moment generating the cortical torsional strains in this orientation. It should be noted that there would also be a “vertical” apical equal and opposite reactive force evenly distributed along the flat face of the block (blue arrows). The measurement of this element was beyond the scope of this experiment. (IAN: inferior alveolar nerve).

This was in congruence with the complex nature of a peri-implant cortico-cancellous bone elastic deformation buttressing effect. This effect served to limit strains to within physiological limits. This is vital to the success of this implant design, which has the combined characteristics of cornered ends (potential stress concentrations) and being intended for the posterior region, where masticatory forces are maximal.

(ii) Vertically. The second ramification of this strain pattern was in the vertical (axial) and bucco-lingual aspect, where there was an overall buccally inclined torsional effect (Figure 8). This was evidenced on the lingual aspect, where there remained high torsional rotation elements (high Φ and ε2), giving a complex picture of lingual compression and a buccally directed torsion.

### 4.3. Mesio-Distal Torsion

**Figure 9:**
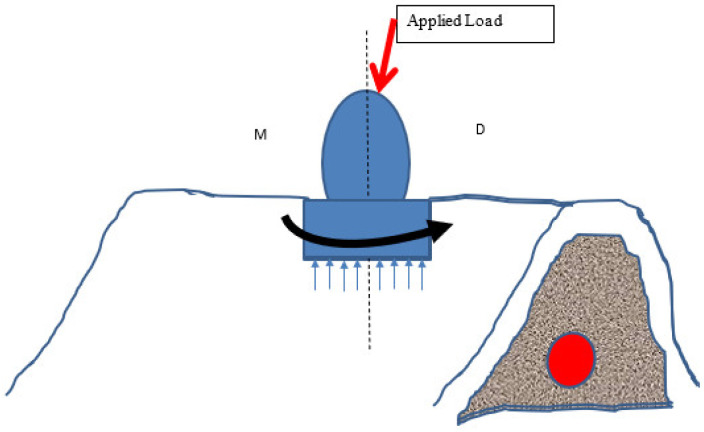
Horizontal bucco-lingual torsional effect: buccal view. The red arrow depicts the applied force, while the horizontal black arrow depicts the “horizontal” mesio-distal resultant moment generating the cortical torsional strains in this orientation. Again, it should be noted that there would also be a “vertical” apical equal and opposite reactive force evenly distributed along the flat face of the block (blue arrows). The measurement of this element was beyond the scope of this experiment, including any of its secondary effects of super-imposing further strains at the positions of the surface strain gauges.

Relatively large ε2 values for both mesial and distal gauges were present. The distal-buccal rotation pattern, as evidenced by the patterns of the principal strains and their angular orientations, was dominant in this (horizontal) plane. That is, the mesial and distal gauges at 1000 N showed opposite directional characteristics, which highlighted a strong counter-clockwise rotation (Figure 9).

Although, from a bucco-lingual aspect, the 30^0^ off axial load would be evident, from the mesio-distal aspect, the force applied at the ledge of the crown should be coincident with the axis of the entire implant crown complex.

It is posited that there were imperceptible off-axial loadings in the mesio-distal engagement of the ledge. This, in turn, would have resulted in a mesio-distal rotational moment generating (via the osseo-integration interface) localized torsional cortical stresses. Such a nonaxial load could result in rotatory cortical torsional stresses, which may be either mesially or distally oriented (clockwise or counter-clockwise).

### 4.4. Combined Mesio-Distal and Bucco-Lingual Torsion

Ultimately, the results of this study have highlighted horizontal and vertical cortical torsional stresses, which, although analyzed separately in the above results, were expressed simultaneously in the peri-implant cortical environment (i.e., the super-imposition of all strains).

**Figure 10:**
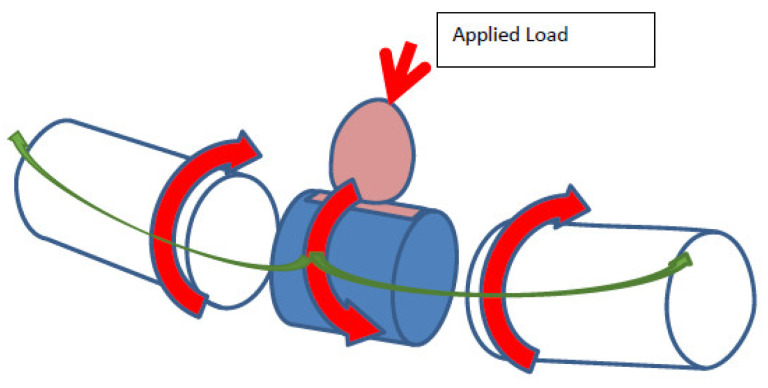
The buccal flexure of the body of the mandible dominated the readings of the buccal gauge.

**Figure 11:**
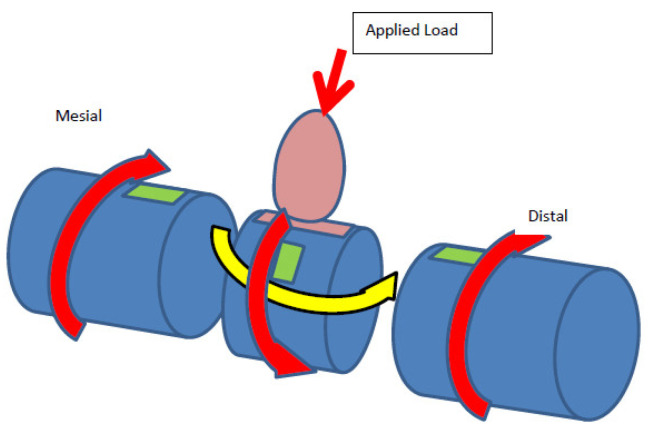
The remaining gauges allowed an overall picture of torsional stresses in the body of the mandible. The horizontally (mesial-distal) oriented rotational moment caused by the inadvertent off-axial load application, as seen from the buccal aspect, has been included as a horizontally oriented torsional load (yellow arrow). In this study, this rotation has been defined as counter-clockwise.

These torsional elements were evident through high ε2 strain gauge values for the lingual, mesial, and distal gauges. It is thought that this was not reflected in the buccal gauge at 1000 N due to the dominant buccal flexure of the body of the mandible at that point (Figure 10). The torsional elements at 1000 N were further evidenced by the complex angular orientations of the principal strains. A diagrammatic representation of the overlay of the horizontal and vertical cortical torsional stresses can be given (Figure 11).

### 4.5. The Complexity of the Principal Strains and their Angular Orientations

The generation of the complex patterns of principal strains and their angular orientations in the results of this study reflected the equally complex relationship between the block implant and its surrounding bony environment. Possible (and undefined) key factors underpinning this complexity included:

(i) The angulation and rotation of the block in the mandible, e.g., the bucco-lingual slant. This factor varied with respect to the differences between the crestal morphology of the alveolar ridge and the underlying cross-sectional morphology of the body of the mandible.
(ii) The particular pattern of osseo-integration around the block itself. Judge [34] extensively studied the trabecular and cortical architecture of the dog mandible, depicting a high level of variation and heterogeneity in its make-up, while Monje et al. [35] have highlighted the variability in trabecular density and volume in the posterior mandible from site to site.
(iii) The particular relative thicknesses of the cortical and cancellous bone, as well as the shape of the mandible at the position of the implant. These factors would have affected the critical elements of cortical flexural stress and cancellous tensile shear stress, as defined by the mathematical principles of such a bi-layered composite beam [36,37].
(iv) The inability to perfectly align the application of the force. The step on the crown and the engaging push rod would inevitably have some degree of variation in their orientations. Deviations from perfect parallelism would be expressed as a bias in force resolution, as well as the resultant axial moments, torsional stress concentrations, and strain levels.

Furthermore, internally within the cancellous bone which forms the bulk of the osseo-integrative connection, the block itself will undergo rotatory influences depending on the exact angulation of the force applied to the prosthetic crown and the alignment of this force system to the implant body and its alignment, in turn, to the body of the mandible.

This complex interplay of both the bucco-lingual cortical and cancellous rotatory moments ultimately expressed influence on the mesial and distal peri-implant bone stresses, producing either compression or tension depending on the rotation experienced. The elucidation of these factors will require further analyses.

In the oral environment, bony architecture is highly variable, opposing teeth may be axially tilted, and there may be little prosthodontic latitude in directing masticatory forces axially. Indeed, it emerges that the current possible paraxial loading and non-parallelism in placement is most likely a reflection of the true clinical setting. There is little information that can clarify these factors, and further analyses beyond the scope of this endeavor are needed.

### 4.6. Physiological Limits

Crucially, the strains generated in the mandible here were within physiological limits [29] (<3000 με, Frost 1987), despite the application of 1000 N of obliquely applied force. The successful ability of the osseo-integrated RBI environment to biomechanically withstand such forces points to two key factors.

(i) **The importance of the role of the mandible as a single and flexible composite beam.**

It was found that, at high loads, tensile strains dominated buccally, while compressive strains dominated lingually. It was posited that these strains were evidence of a buccal-lingual mandibular torsional flexure, both in the vertical and horizontal planes.

The differential in the moduli of elasticity between the cortical and cancellous osseous components would yield a rigid yet flexible beam. The successful osseo-integration of the block into the mandible meant that it was essentially a fixed component of this “beam”, and the forces applied to the implant were transferred to the body of the mandible itself. The predominant stresses generated were buttressed through both cancellous bone shear and cortical axial bending stresses.

Despite having a higher failure rate than their longer counterparts, short implants have been extensively documented as being successful, even with crown-to-implant ratios exceeding 200%. It is postulated that the observations of this component on a more general level elucidate a fundamental factor at play, which, although present in all implants, is especially highlighted in short implants; the higher concentration of stresses per unit area of the implant is dissipated through the flexure of the mandible itself, acting as a single composite beam. What emerges is a reduction in peri-implant strain levels to within physiological limits, even when forces are applied that would be expected to produce strains beyond physiological limits. These results are in congruence with the results of:

Kan et al. [2]: masticatory overloaded implant scenarios failed to yield peri-implant stresses beyond physiological limits.

Judge [34]: masticatory mechanical loading of the mandible yielded flexure and stress-induced elastic deformation of the mandible as a whole, as evidenced by a complex array of transient strains, even at sites distant to the implant.

It must be highlighted that this study has focused solely on the use of cortically placed strain gauges. Cancellous bone tensile strength is also a critical factor in mandibular composite beam analysis. Assessment of this, however, requires the trabecular assessment of strains. Although beyond the scope of this analysis, it emerges that further studies are needed using trabecular, as well as cortical bone strain gauge readings.

(ii) **The rectangular design.**

It is posited that the flat walls of the polygonal block design acted to reduce torsional stress concentrations by providing a large wall-like flat surface area that served to yield a more even distribution of stresses. In the buccal “vertical” torsional stress orientations, this was achieved by the flat 24 mm^2^ face of the block. This is to be contrasted with the uneven stress distribution of a cylindrical counterpart, where the most buccal radial curvature must reflect the greatest burden of stress concentration.

**Figure 12:**
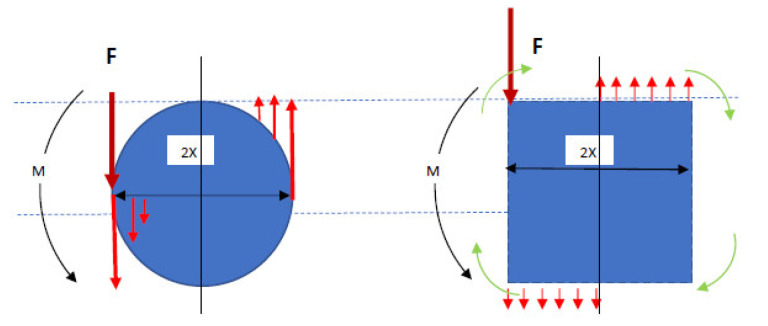
Applied horizontal rotatory force: occlusal view of a cylindrical implant of diameter 2X and a block implant of length 2X: an equal moment M, is generated through an equal force F, applied at an equal distance X from the center of each structure. It is seen that the torsional surface stresses will be more concentrated at the radial tangents of the cylinder compared to the lower, more even distribution of the torsional loads of the flat-faced block (depicted as red arrows). The corners of the block wound also act in opposing resistive directions to the applied moment (green arrows), further reducing the overall peri-implant torsional stresses.

This difference was also reflected in the mesio-distal “horizontal” torsional stress orientation. The radially symmetrical nature of a cylinder will induce higher radial torsional stresses within the investing bone at its mesial and distal tangents when subjected to a rotatory load as compared to a rectangular block of the same mesio-distal maximal dimension (Figure 12). This is, in part, due to the anti-rotational geometry of the block resulting from its length and its corners and cornered edges. These factors will also act to reduce peri-implant torsional stresses of the osseo-integrated RBI.

### 4.7. Limitations

It is acknowledged that the results of this study must be interpreted with caution, as it is lacking statistical power. Further studies are needed, where larger samples of implant bone samples are used to yield high numbers of results. The utilization of strain gauges [19,34] in these samples would also yield data for future FEA of the osseo-integrated RBI. These limitations were underpinned by both financial and ethical approval constraints. These constraints impacted directly on the number of implants and animals used, and, hence, the lack of controls, as well as the small sample size. 

It is further conceded that this study is animal-based; hence, caution should be imposed when extrapolating implications of these results to the human situation.

## 5. Conclusions

The observations of this limited qualitative analysis showed that the applied off-axial load to the osseo-integrated RBI generated a heterogeneous pattern of strains. The dominant characteristics of this pattern were:

(i) Tensile and compressive strains were expressed bucco-lingually and mesio-distally.
(ii) Bucco-lingually, there was a “horizontal” flexure of the body of the mandible, producing buccal tensile strains and lingual compressive strains.
(iii) Bucco-lingually, there were strong “vertical” torsional stresses directed apically on the buccal aspect.
(iv) Mesio-distally, there were strong horizontal torsional stresses in a disto-buccal orientation.

The short dimensioned osseo-integrated RBI design appeared to biomechanically withstand the deliberately exaggerated off-axial simulated masticatory loads and to maintain the strains generated to levels that were within physiological limits. The conclusions and ramifications of this study are provisional, as more studies and statistical analyses are needed to confirm them.

## Figures and Tables

**Table 1:** The overall mean amplitudes of each gauge output were derived as the mean difference between the maxima and minima of each channel.

Mesial, Buccal, Distal, and Lingual Gauges (All Readings in Micro-Strain: με)				
	Channel	ε1	ε2	ε3
	**mesial gauge**			
	mean max-min difference	798 ± 35	−1551 ± 98	−639 ± 47
	**tens/comp**	tension	compression	compression
**1000 N**				
	**buccal gauge**			
	mean max-min difference	−1029 ± 104	489 ± 23	1618 ± 121
	**tens/comp**	compression	tension	tension
	**dis** **tal gauge**			
	mean max-min difference	−285 ± 31	−1281 ± 127	−1742 ± 186
	tens/comp	compression	compression	compression
**1000 N**				
	**lingual gauge**			
	mean max-min difference	−707 ± 56	732 ± 17	757 ± 98
	tens/comp	compression	tension	tension

**Table 2:** Principal strains and their orientations. It is clarified here that, although the positive and negative values of the principal strains refer to tensile and compressive strains, respectively, the overall understanding of the directions of the strains is gained when the angular orientation is factored-in.

Principle Strains: Magnitudes (με) and Angular Resolve					
Load	Gauge	εp	εq	θ/φ^0^	Absolute Value
	Mesial	1822	−1663	33.0	ε1 > ε3 (ε2 large)
**1000 N**	Distal	67	−2094	10.0	ε1 > ε3 (ε2 large)
	Buccal	1632	−1043	4.2	ε1 < ε3
	Lingual	2096	−2047	21.9	ε1 > ε3 (ε2 large)

## Data Availability

No new data were created or analyzed in this study. Data sharing is not applicable to this article.
